# Isolation, Identification, and Characterisation of Degradation Products and the Development and Validation of a Stability-Indicating Method for the Estimation of Impurities in the Tolterodine Tartrate Formulation

**DOI:** 10.3797/scipharm.1407-18

**Published:** 2014-09-08

**Authors:** Lakkireddy Prakash, Malipeddi Himaja, Rudraraju Vasudev

**Affiliations:** 1 Department of Analytical Research and development, Dr. Reddy’s, Laboratories Ltd, IPDO, Hyderabad, 500072, Telangana, India; 2 Pharmaceutical Chemistry Division, School of Advanced Sciences, VIT University Vellore-632014, TN, India

**Keywords:** Tolterodine tartrate, Stability-indicating, Development, Characterisation, UPLC

## Abstract

A short and sensitive stability-indicating gradient RP-UPLC method was developed for the quantitative determination of process-related impurities and degradation products of tolterodine tartrate in pharmaceutical formulations. The method was developed by using the Waters ACQUITY UPLC™ BEH shield RP18 (2.1 × 100 mm, 1.7 μm) column with a mobile phase containing a gradient mixture of solvent A and B at a detection wavelength of 210 nm. During the stress study, the degradation products of tolterodine tartrate were well-resolved from tolterodine and its impurities and the mass balances were found to be satisfactory in all the stress conditions, thus proving the stability-indicating capability of the method. The developed method was validated as per ICH guidelines with respect to specificity, linearity, limit of detection and quantification, accuracy, precision, ruggedness, and robustness. During the stability (40°C/75% RH, 3 months) analysis of the drug product, one unknown impurity was detected by the above stability-indicating method. The unknown impurity was isolated by preparative HPLC and subjected to mass and NMR studies. Based on the spectral data, the unknown impurity was characterised as 2-(3-amino-1-phenylpropyl)-4-methylphenol (des-*N*,*N*-diisopropyl tolterodine). Structural elucidation of the impurity by spectral data is discussed in detail.

## Introduction

Tolterodine tartrate (2-{(1*R*)-3-[di(propan-2-yl)amino]-1-phenylpropyl}-4-methylphenol 2,3-dihydroxybutanedioic acid (1:1) salt, C_22_H_31_NO•C_4_H_6_O_6_) is an antimuscarinic (muscarinic receptor antagonist) drug that is used to treat overactive bladder and symptoms associated with voiding such as urge urinary incontinence, urgency, and frequency. It controls bladder incontinence by controlling contractions [1–3]. It acts by competitively antagonizing muscarinic receptors, inhibiting bladder contractions, and reducing urinary frequency. It comes under the pharmacologic class: anticholinergic and the therapeutic class: urinary tract antispasmodic.

The literature survey revealed various spectrophotometric methods [4, 5], stability-indicating HPLC methods for the quantification of tolterodine [6–9], and in plasma [10–13] the dosage forms have been reported. An enantio-specific HPLC method for the determination of (S)-enantiomer impurities in (R)-tolterodine tartrate [14], a validated chiral HPLC method for the separation of enantiomers [15], and HPLC methods for the determination of related substances of tolterodine tartrate [16, 17] have been reported. The isolation and identification of degradation products of tolterodine tartrate tablets were also reported [18, 19]. However, reported methods have not mentioned the formation of a new degradant, des-*N*,*N*-diisopropyl tolterodine. This unknown impurity was detected in the drug product during stability sample analysis, which crossed the identification threshold. As per the stringent regulatory requirements recommended by the ICH and regulatory agencies, it is mandatory and important to identify and structurally characterize any impurity formed during production and stability testing, exceeding the identification threshold [20–24]. Various analytical instruments and advanced hyphenated techniques [25–36] are routinely used to carry out the impurity profile study.

The degradation product was isolated by preparative HPLC and subjected to ESI-MS/MS, UPLC-TOF MS, and ^1^H and ^13^C NMR spectral studies. Based on the spectral data, the unknown impurity was characterized as N, N des diisopropyl tolterodine. To the best of our knowledge, this impurity has not been reported elsewhere. In the literature, there is no stability-indicating LC method available for the estimation of N, N des diisopropyl tolterodine in pharmaceutical formulation. The present study describes the isolation and characterisation of N,N des diisopropyl tolterodine, as well as the development and validation of a stability-indicating RP-UPLC method for the estimation of degradation and process-related impurities of tolterodine tartrate, namely N,N des diisopropyl tolterodine, impurity (imp) A, imp B, imp C, imp D, and imp E (Table 1). Forced degradation studies were performed on the placebo (all excipient mixtures without the tolterodine tartrate drug substance) and the drug product to show the stability-indicating nature of the method. These studies were performed in accordance with established ICH guidelines [37, 38].

**Tab. 1:**
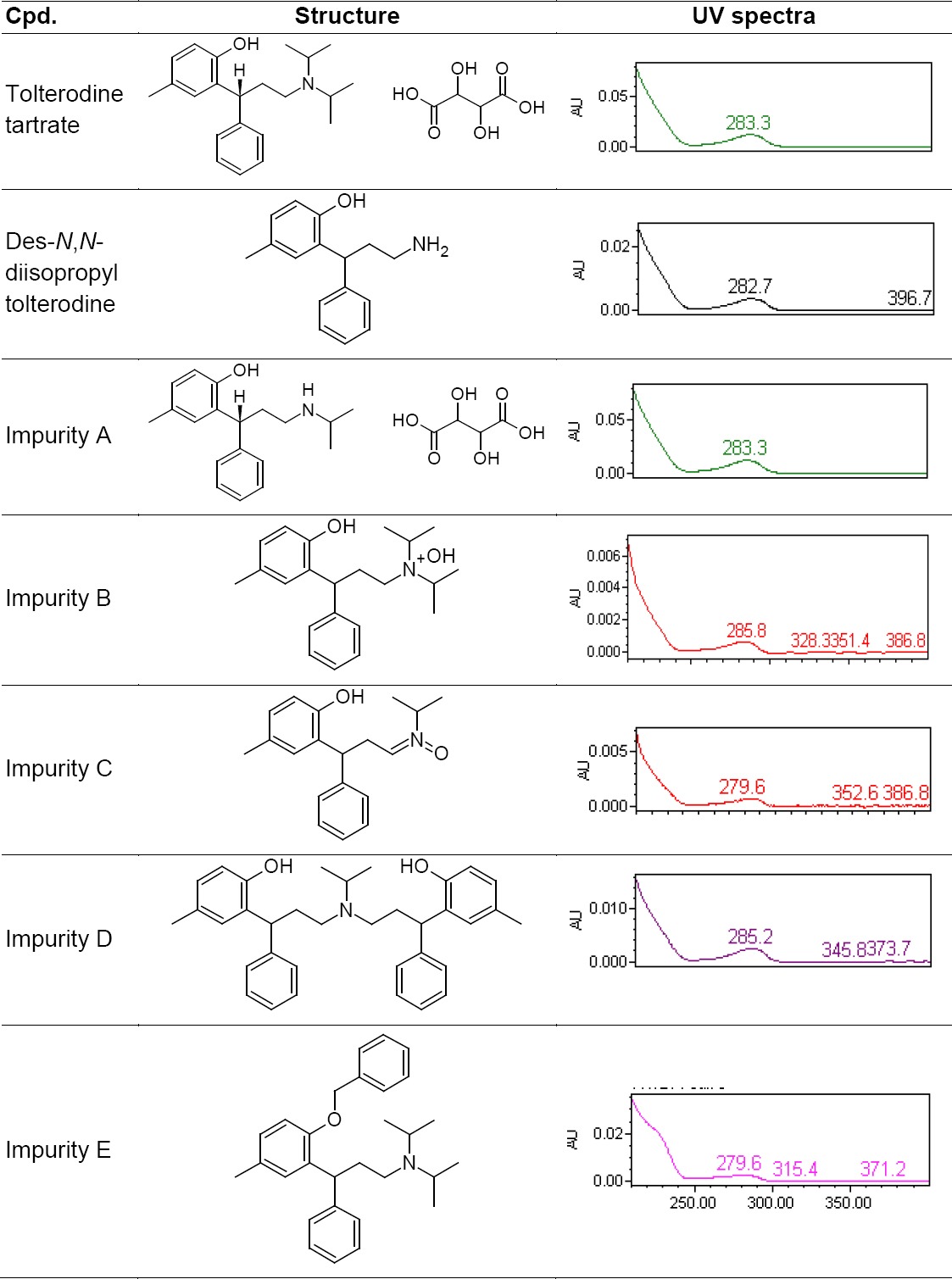
Names, structures, and UV spectra of tolterodine tartrate and its impurities

## Experimental

### Chemicals and Reagents

The tolterodine tartrate drug product and its impurities were obtained from Dr. Reddy’s Laboratories Limited, Hyderabad, India. The HPLC grade acetonitrile, analytical grade KH_2_PO_4_, trifluoro acetic acid, and orthophosphoric acid were purchased from Merck, Mumbai, India. High-purity water was collected from a Millipore Milli-Q Plus water purification system (Millipore, Milford, MA, USA). DMSO-d_6_ (for NMR) was obtained from Aldrich Chemical Co.,USA.

### Equipment

The Acquity UPLC^™^ (Water, Milford, USA) was used which is equipped with a binary solvent manager, a sample manager, and a photodiode array (PDA) detector. The output signals were monitored and processed using Empower 2 software. The Cintex digital water bath was used for the hydrolysis studies. Photostability studies were carried out in a photostability chamber (Sanyo, Leicestershire, UK). Thermal stability studies were performed in a dry air oven (Cintex, Mumbai, India). The pH of the solutions was measured by a pH meter (Mettler-Toledo, Switzerland).

### Chromatographic Conditions

The method was developed using an ACQUITY UPLC™ BEH shield RP18 (2.1 × 100 mm, 1.7 μm) column with a mobile phase containing a gradient mixture of solvent A (0.01 M potassium dihydrogen phosphate, pH-adjusted to 3.5 with orthophosphoric acid) and B (900:100 mL mixture of acetonitrile and solvent A). The gradient program (time (min) / %B) was set as 0/45, 2/53, 2.5/80, 3/95, 5/100, 5.1/45, and 7/45. The mobile phases were filtered through nylon 0.45 μm membrane filters and degassed. The flow rate of the mobile phase was 0.4 mL/min. The column temperature was maintained at 50°C and the eluted compounds were monitored at the wavelength of 210 nm. The injection volume was 2.0 μL. The optimized liquid chromatography (LC) conditions are described in Table 2.

**Tab. 2:**
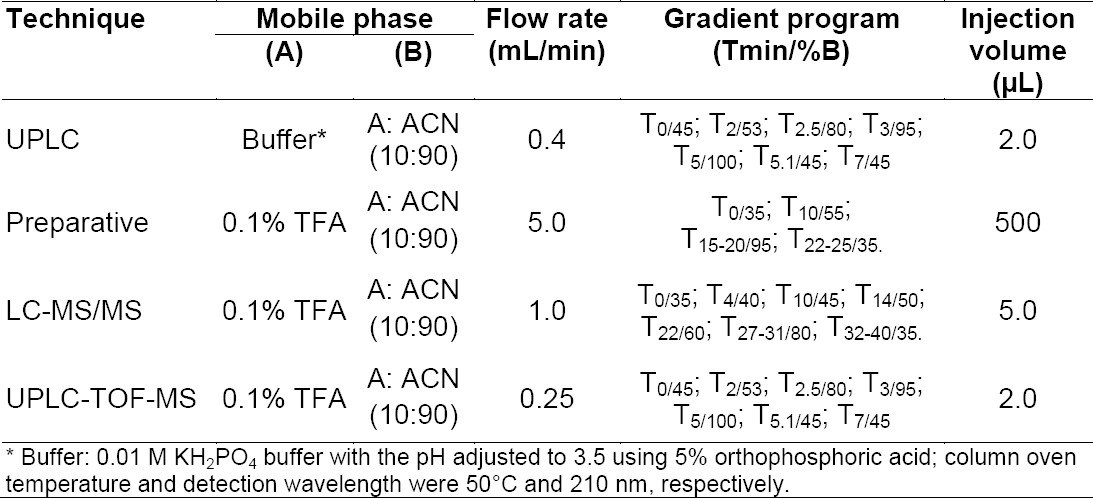
LC conditions for analytical, preparative, LC-MS/MS, and UPLC-TOF-MS analyses

### Preparative HPLC Conditions

Preparative isolation work was carried out on an Agilent 1200 series preparative HPLC system which is equipped with an automated fraction collector and photodiode array detector. The data was monitored and processed using Chemstation software. Mobile phase A contained 0.1% TFA in water and mobile phase B contained a mixture of mobile phase A and acetonitrile in the ratio of 10:90 (v/v). An XBridge™ Prepshield RP 18 (10 × 250 mm, 5.0 μm) column was employed for the isolation of the unknown impurity. Approximately 15 mg/mL of the sample was prepared to load onto the column. The gradient program (time (min) / %B) was set as 0.01/35, 10/55, 15/95, 20/95, 22/35, and 25/35 with a flow rate of 5.0 mL/min and injection volume of 1000 µL. The column temperature was maintained at 40°C and the peaks were monitored at 210 nm. A mixture of mobile phase A and acetonitrile in the proportion of 50:50 (v/v) was used as the diluent for the sample preparation. The optimized preparative LC conditions are described in Table 2. The impurity fractions were collected separately from several injections and pooled together. The pooled fraction was concentrated by using the Rotavapor (model: Heidolph Laboratory 4002 control) under high vacuum. The aqueous solutions were lyophilized (model: Virtis Advantage Plus) to solidify the impurity.

### LC–MS/MS Conditions

The electrospray ionization and MS-MS studies were performed on the triple quadrupole mass spectrometer PE Sciex Model: API 3000. The positive and negative electrospray MS data were obtained by switching the capillary voltage between +5000 and -4500, respectively. The MS-MS data were generated with collision energy ramping from 30-60 volts in nitrogen atmosphere. A mixture of water and methanol in the proportion of 50:50 (v/v) was used as diluent for the sample preparation and the concentration of the sample was 0.02 mg/mL. An ACE C18, (250×4.6 mm, 5 μm) column was used for separation. The optimized LC conditions are described in Table 2.

### UPLC-TOF-MS Conditions

The UPLC-TOF-MS system consisted of an ACQUITY^™^ Ultra Performance Liquid Chromatography (UPLC) system and a Micromass LCT Premier XE Mass Spectrometer (high sensitivity orthogonal time-of-flight instrument, Waters, Milford, USA) equipped with a lock mass sprayer, operating in either positive or negative ion mode. All analyses were acquired using the lock spray to ensure accuracy and reproducibility; leucine enkephalin was used as the lock mass. High resolution (W mode, FWHM 10500) positive polarity scan responses were collected from m/z 100 to 1000 at a rate of 1.0 s/scan. The chromatographic column used was an ACQUITY UPLC™ BEH shield RP18 (2.1 × 100 mm, 1.7 μm). Mobile phase A contained 0.1% TFA and mobile phase B contained a mixture of 0.1% TFA and acetonitrile in the ratio of 10:90 (v/v), respectively. The gradient program (time (min) / %B) was set as 0/45, 2/53, 2.5/80, 3/95, 5/100, 5.1/45, and 7/45 with a flow rate of 0.4 mL/min and injection volume of 2.0 µL. The mobile phases were filtered through nylon 0.45 mm membrane filters and degassed. The column temperature was maintained at 50°C and the peaks were monitored at 210 nm. The optimized LC conditions are described in Table 2. A mixture of water and methanol in the proportion of 50:50 (v/v) was used as diluent for sample preparation.

### NMR Spectroscopy

NMR experiments were performed using a 500 MHz Unity INOVA NMR spectrometer (Varian) in DMSO-d_6_ at 25°C as the solvent. ^1^H NMR measurements were carried out at 500 MHz, while ^13^C NMR experiments were performed at 125 MHz. Proton and carbon chemical shifts were reported on the δ scale in ppm, relative to tetramethylsilane (TMS) (δ= 0.00 ppm) and DMSO (δ = 39.50 ppm) as internal standards in ^1^H and ^13^C NMR spectra, respectively. Standard pulse sequences provided by Varian were used for 1D and 2D NMR data.

### Preparation of Solutions

#### Preparation of Diluent

A mixture of analytical solvent A:acetonitrile in the ratio 50:50(V/V) was used as the diluent.

#### Preparation of the Standard Solution

Stock solution of tolterodine tartrate was prepared in the diluent with a concentration of 300 μg/mL. Working standard solution was prepared from diluting the above stock solution of tolterodine tartrate with a final concentration of 1.5 μg/mL.

#### Preparation of the Sample Solution

With an equivalent amount to 25 mg of tolterodine tartrate, the drug product was dissolved in diluent with sonication for about 30 min to prepare a solution containing 500 μg/mL of the drug. This solution was centrifuged at 10000 rpm for about 10 min.

### Method Validation

The proposed method was validated as per ICH guidelines [39]. The following validation characteristics were addressed: specificity, accuracy, precision, limit of detection, limit of quantification, linearity, range, ruggedness, and robustness.

#### System Suitability

System suitability was determined before the sample analysis from six replicate injections of the standard solution containing 1.5 μg/mL of the drug. The acceptance criteria were less than 5% of the relative standard deviation (RSD) for the peak areas, and the USP tailing factor less than 2.0 for the tolterodine peak from the standard solution.

#### Specificity/Stress Studies

Specificity is the ability of the method to measure the analyte response in the presence of its potential impurities. The specificity of the developed LC method for tolterodine tartrate was carried out in the presence of its impurities and degradation products. Stress studies were performed at the 500 μg/mL concentration of tolterodine tartrate on the drug product to provide an indication of the stability-indicating property and specificity of the proposed method. The stress conditions employed for the degradation study included acid hydrolysis (1 N HCl at 80°C for 2 hours), base hydrolysis (1 N NaOH at 80°C for 2 hours), oxidation (6% H2O2 at 50°C for 2 hr), hydrolytic (water at 80°C for 2 hr), thermal (105°C for 24 hr), and photolytic degradation (drug product exposed to visible light for 240 hr resulting in an overall illustration of 1.2 million lux hours and UV light for 250 hr resulting in an overall illustration of 200 watt hours/m^2^ at 25°C). A peak purity test was carried out for the tolterodine peak by using the PDA detector in the stress samples. Placebo interference was evaluated by analysing the placebo prepared as per the test method.

#### Precision

The precision of the method was verified by repeatability and intermediate precision. Repeatability was checked by injecting six individual preparations of tolterodine tartrate drug product spiked with its six impurities; N,N des diisopropyl tolterodine, imp A, imp B, imp C, imp D, and imp E at the 0.30% level (0.30% of impurities with respect to 500 µg/mL tolterodine tartrate). The RSD (%) of the area for each impurity was calculated. The intermediate precision of the method was also evaluated using a different analyst and a different instrument and performing the analysis on different days.

#### Limits of Detection (LOD) and Quantification (LOQ)

The LOD and LOQ for tolterodine tartrate and its impurities were determined at a signal-to-noise ratio of 3:1 and 10:1, respectively, by injecting a series of dilute solutions with known concentrations. The precision study was also carried out at the LOQ level by injecting six individual preparations of tolterodine tartrate and its impurities and calculating the %RSD of the area.

#### Linearity

Linearity test solutions were prepared by diluting the stock solutions to the required concentrations. The solutions of each impurity were prepared at six concentration levels from the LOQ to 200% of the specification level. Calibration curves were plotted between the responses of the peak versus the analyte concentrations. The coefficient correlation, slope, and y-intercept of the calibration curve were reported.

#### Accuracy

Standard addition and recovery experiments were conducted on the real sample (drug product) to determine the accuracy of the related substance method. The accuracy of the method for tolterodine tartrate, N,N des diisopropyl tolterodine, imp A, imp B, imp C, imp D, and imp E was evaluated in triplicate using six concentration levels of the LOQ, 50%, 75%, 100%, 125%, and 150% of the target concentration level. The percentage recoveries for each impurity were calculated.

#### Robustness

To determine the robustness of the developed method, experimental conditions were deliberately altered and the system suitability parameters for the tolterodine tartrate standard were recorded. The variables evaluated in the study were the pH of the mobile phase buffer (± 0.2), column temperature (± 5°C), flow rate (± 0.02 ml/min), and % organic solvent in the mobile phase (± 10%).

#### Solution Stability and Mobile Phase Stability

The solution stability of tolterodine tartrate and its impurities was determined by keeping the test and standard solutions in tightly capped volumetric flasks at room temperature for up to 48 hours and measuring the amount of the six impurities at every 24 hour interval against freshly prepared standard solution. The mobile phase stability was also determined by injecting freshly prepared solutions of tolterodine tartrate and its impurities at 24 hours and 48 hours. The mobile phase was not changed during the study.

## Results and Discussion

### Structure Characterization of the Unknown Impurity

An unknown impurity with a relative retention time (RRT) of 0.52 with respect to tolterodine was observed during the stability (40°C/75% RH, 3 months) study of the drug product and we tried to enhance the impurity by using the forced degradations to isolate it. But the impurity was not increased in any trial. So the impurity was isolated by semi-preparative HPLC from stability samples with a purity of > 97% and used for its characterisation by LC-MS and NMR studies.

**Fig. 1:**
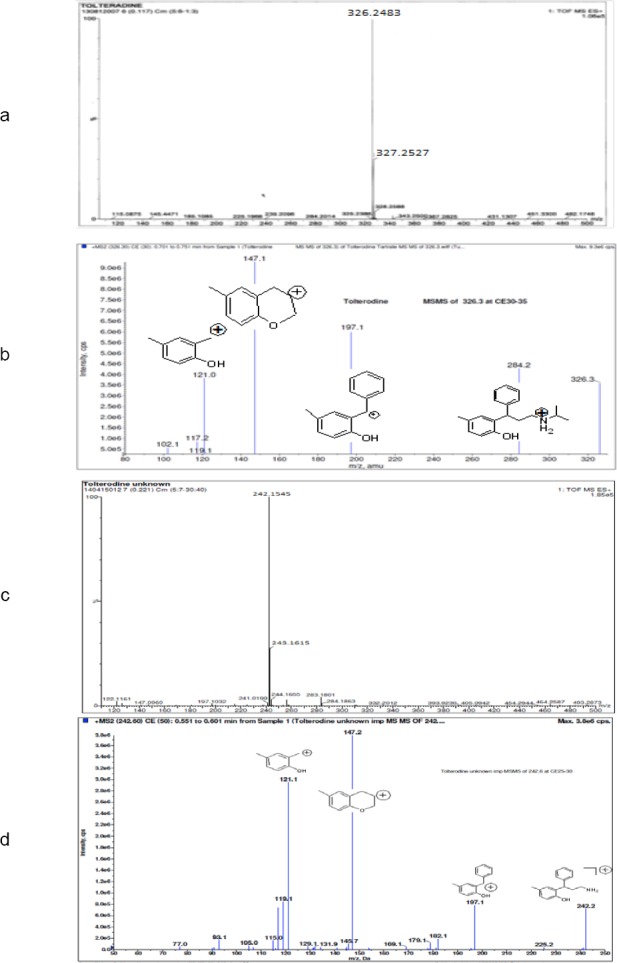
HR-MS and MS–MS data of tolterodine and the unknown impurity (a) HR-MS spectra of tolterodine, (b) MS–MS spectra of tolterodine, (c) HR-MS spectra of the impurity, (d) MS–MS spectra of the impurity

The positive ESI-MS spectrum of the unknown impurity showed a peak at m/z 242.2 amu [M+H]^+^ (Fig. 1d) which was 84 amu less than that of tolterodine (m/z 326).The positive HR-MS spectrum showed a protonated molecular ion peak at m/z 242.1545 (Fig.1c) corresponding to the molecular formula C_16_H_20_NO. In comparison with tolterodine, the unknown impurity has six fewer carbon and twelve fewer hydrogen atoms. This can be attributed to the loss of two isopropyl groups. The comparison of MS/MS studies of the unknown impurity and tolterodine showed common fragment ions at m/z 197.1, 147.1, and 121.1 (Fig. 1b & 1d). The common fragment ion peak suggests that 2-benzyl-4-methylphenol was intact and changes were at the nitrogen atom.

**Fig. 2:**
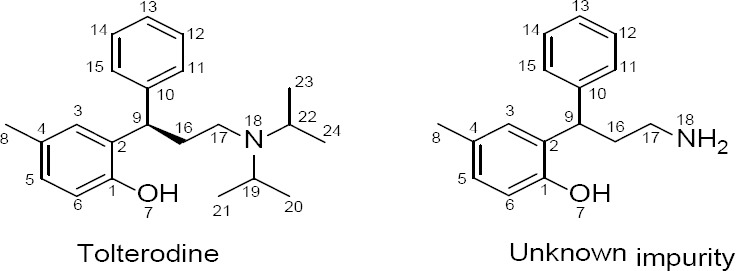
Chemical structures of tolterodine and the unknown impurity with numbering

**Fig. 3:**
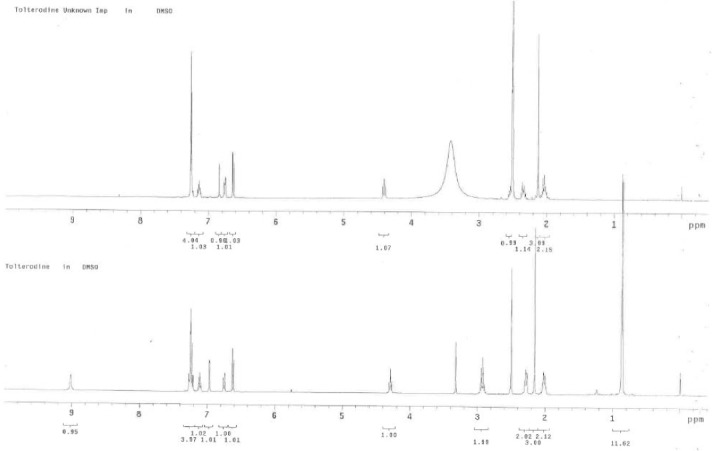
Overlay of the NMR spectra of the unknown impurity and tolterodine

To get the structural information, the isolated product was further subjected to the ^1^H and ^13^C NMR study. The number of proton and carbon resonances is less than that in tolterodine. The variation in chemical shifts was observed in both ^1^H and ^13^C at methylene (17th position) for the hydrogens (shifted slightly toward downfield) and the carbons (shifted slightly toward up field) present on the aliphatic side chain attached to the nitrogen atom (Table 3) when compared to that of tolterodine. It indicates that there should be changes at the nitrogen atom (18th position). The comparison of the ^1^H NMR spectra of the unknown impurity and tolterodine (Fig. 3) showed the absence of isopropyl groups. The absence of carbon signals corresponding to the two isopropyl moieties in the ^13^C NMR spectrum supports the loss of two isopropyl groups from tolterodine and the formation of the degradant. This is in agreement with the HRMS pattern observed for the unknown impurity. Based on the above spectral data, the structure of the unknown impurity was characterized as 2-(3-amino-1-phenylpropyl)-4-methylphenol (des-*N*,*N*-diisopropyl tolterodine). The structures are shown in Fig. 2.

**Tab. 3:**
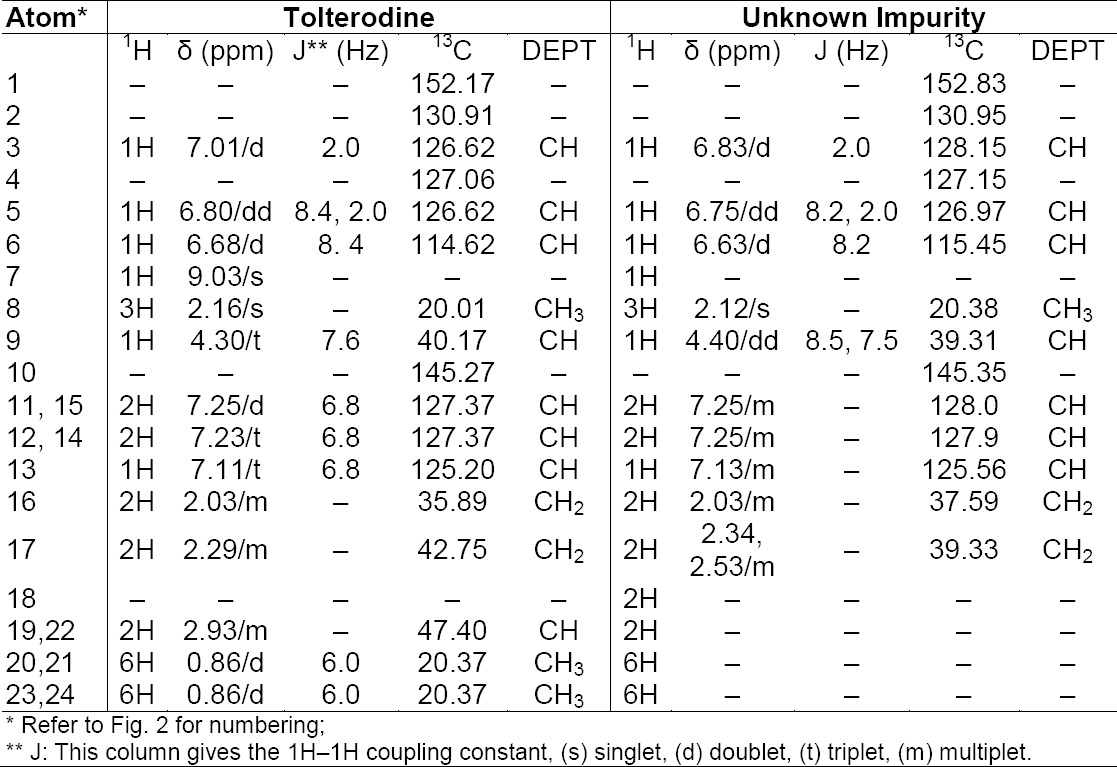
^1^H and ^13^C NMR assignments for tolterodine and the unknown impurity

### Method Development and Optimization of the Stability-Indicating UPLC Method

The main objective of the chromatographic method was to develop a stability-indicating UPLC method that separates the active ingredient without the interference from degradation products, process impurities, excipients, or other potential impurities and accurately measures all the related impurities with a shorter run time. Preliminary experimental conditions were set on the basis of information about physicochemical properties of the drug substance like the dissociation constant, partition coefficient, chromatographic behaviour, spectrophotometric properties, and published methods available in the literature. Individual stock solutions of tolterodine tartrate and its impurities were injected and we checked the spectra of each component. From the spectral data, the maximum absorbance was observed between the wavelengths of 278 nm to 284 nm, but the response was found to be much less between these wavelengths. The response was found to be high for all the impurities and tolterodine at 210 nm. Hence, 210 nm was selected for the estimation of tolterodine and its impurities.

**Fig. 4:**
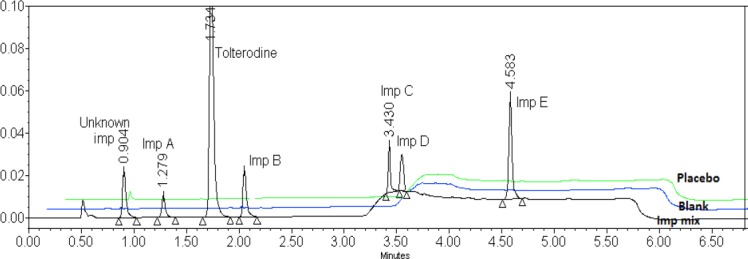
Typical overlay chromatogram of the blank, placebo, and impurity mixture

The blend containing 500 μg/ml of tolterodine tartrate and 1.5 μg/ml of each imp A, imp B, imp C, imp D, imp E, and des-*N*,*N*-diisopropyl tolterodine was used for separation. By thorough study of the literature with an objective to develop a novel and short UPLC method, separation was attempted on the Acquity BEH C18 (50 mm X 2.1 mm, 1.7 μm) column using solvent A (0.01 M potassium dihydrogen phosphate in aqueous solution with a pH of 3.50 and solvent B (buffer and acetonitrile in 20:80 v/v) in different gradient programs at a flow rate of 0.25 mL min^−1^ on UPLC equipped with a photodiode array detector. Under these conditions all the peaks were well-separated but one blank peak was interfering with imp A and there was a huge drift in the base line. To clear the blank peak interference with imp A and to stabilize the gradient drift, an attempt was made with a modified gradient program, a change in solvent B composition, and the use of different columns like the Acquity BEH C8 (100 mm X 2.1 mm) 1.7 μm, Acquity HSS C18 (100 mm X 2.1 mm) 1.8 μm, and Acquity BEH C18 shield (100 mm X 2.1 mm) 1.7 μm columns with a flow rate of 0.4 mL/min. Under the studied chromatographic conditions, in the first two columns, the API peak and imp B, imp C, and imp D were merging. Tolterodine and all six impurity peaks were well-resolved from each other and from the degradation products on the Acquity BEH C18 shield (100 mm X 2.1 mm) 1.7 μm only. A summary of column optimization trials are presented in Table 4. Based on these experiments, gradient program optimization trials were taken on the Acquity BEH C18 shield (100 mm X 2.1 mm) 1.7 μm with the aim to cut down the run time to as minimum as possible. The final optimized conditions are described in Table 2. All the impurities were well-separated with a resolution greater than 1.5. No chromatographic interference due to the blank (diluent) and other excipients (placebo) at the retention time of tolterodine and its impurities was observed. The typical overlay chromatogram of the blank and placebo spiked test is shown in Fig. 4. Degradation samples were injected in the optimised method to check the interference of degradation products with tolterodine and the mass balance of the product after the degradation study. The peak purity of tolterodine was unaffected by the presence of its impurities, degradation products, and other excipients (placebo) which thus confirms the stability-indicating power of the developed method. The mass balance results were found to be more than 97.0%.

**Tab. 4:**
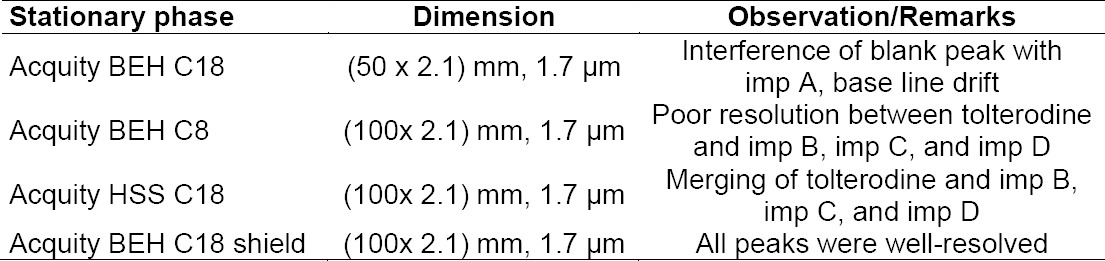
Summary of the stationary phase used to optimize the method

### Method Validation

The proposed method was validated as per ICH guidelines. The method was validated to demonstrate that it is suitable for its intended purpose by the standard procedure to evaluate the adequate validation characteristics (system suitability, specificity, accuracy, precision, linearity, robustness, ruggedness, solution stability, LOD and LOQ, and stability-indicating capability).

#### System Suitability

System suitability shall be checked for the conformance of suitability and reproducibility of the chromatographic system for analysis. The system suitability was evaluated on the basis of resolution, RSD (%) of the peak area, and USP tailing factor for the tolterodine peak from the standard solution; all critical parameters tested met the acceptance criteria (Table 5).

**Tab. 5:**
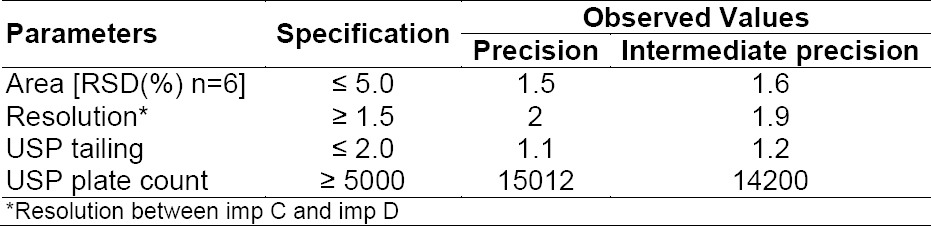
System suitability test results

#### Specificity

The aim of the specificity study was to unequivocally assess the analyte in the presence of components (impurities and degradants) that may be expected to be present. Placebo interference was evaluated by analysing the placebo prepared as per the test method. No peak due to the placebo was detected at the retention time of tolterodine and its impurities. All the forced degradation samples were prepared at a concentration of 500 μg/mL and analysed using a PDA detector to ensure the homogeneity and purity of the tolterodine peak. Significant degradation was observed under base hydrolysis (1 N NaOH at 80°C for 2 h) and slight degradation was observed under oxidation degradation (6% H2O2 at 50°C for 2 h) and thermal degradation (105°C for 24 h). Tolterodine was found to be stable under acid hydrolysis (1 N HCl at 80°C for 2 h), hydrolytic (water at 80°C for 2 h), humidity (25°C/90% RH for 7 days), and photolytic (exposed to 1.2 million lux h visible light and 200 watt h/m^2^ UV light) degradation conditions. The mass balance (% assay + % sum of all degradants + % sum of all impurities) results were calculated and found to be more than 97.96% (Table 6). The purity of tolterodine was unaffected by the presence of its impurities, degradation products, and other excipients (placebo) and thus confirms the stability-indicating power of the developed method.

**Tab. 6:**
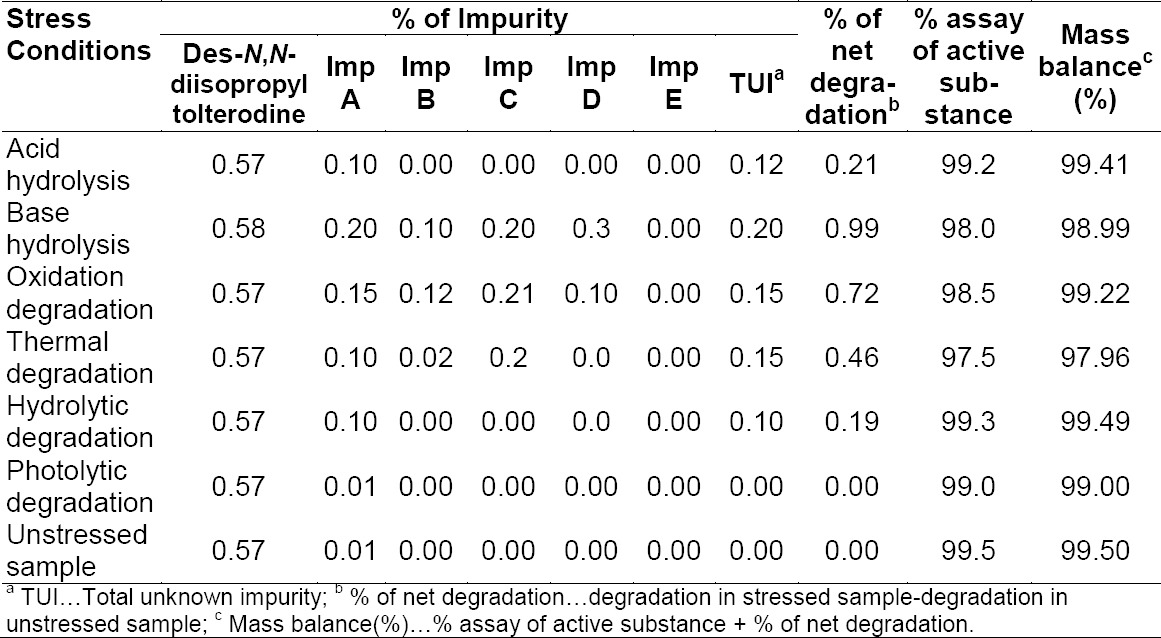
Summary of forced degradation results

#### Precision

The RSD (%) for the area of tolterodine, des-*N*,*N*-diisopropyl tolterodine, imp A, imp B, imp C, imp D, and imp E in the repeatability study was within 1.5% and in the intermediate precision study it was within 1.6%, which confirms the good precision of the method. The RSD (%) values are presented in Table 7.

#### Limits of Detection and Quantification

The limit of detection, limit of quantification, and precision at the LOQ values for tolterodine, des-*N*,*N*-diisopropyl tolterodine, imp A, imp B, imp C, imp D, and imp E are reported in Table 7.

**Tab. 7:**
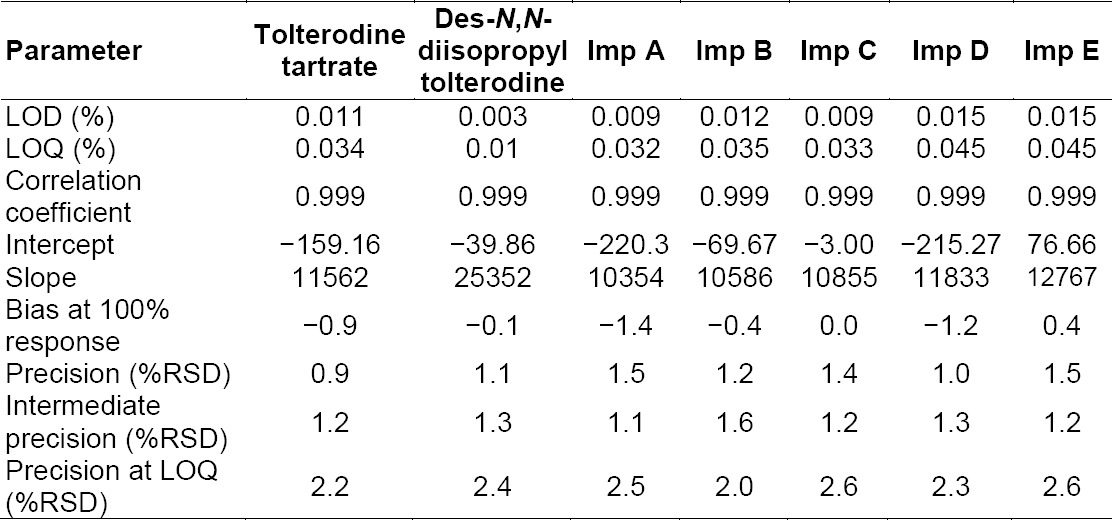
LOD, LOQ, linearity, and precision data

#### Linearity

The linearity calibration plots for tolterodine, des-*N*,*N*-diisopropyl tolterodine, imp A, imp B, imp C, imp D, and imp E were obtained over the calibration ranges tested, i.e. the LOQ to 200% of the specification level. The correlation coefficient obtained was greater than 0.998 and the % bias at 100% response was within 5% (Table 7), proving that a strong correlation exists between the peak area and concentration of tolterodine and its impurities.

#### Accuracy

The percentage of recoveries for tolterodine and its six impurities ranged from 99.5 to 102.5%. The results are reported in Table 8.

**Tab. 8:**
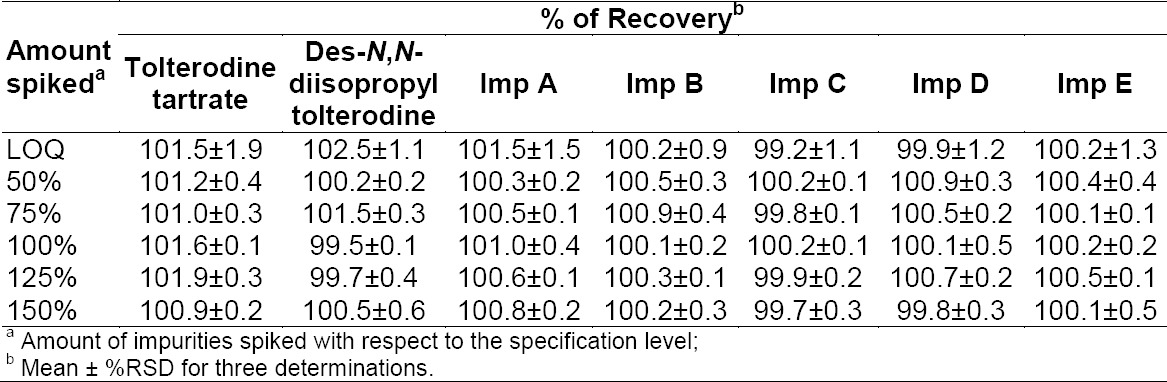
Recovery data

#### Robustness

In all of the deliberately varied chromatographic conditions (flow rate, column temperature, pH of the mobile phase buffer, and composition of the organic solvent), all analytes were adequately resolved and the elution order remained unchanged. The resolution between the critical pair, i.e. for imp C and imp D, was greater than 1.5 and the tailing factor for the tolterodine peak from the standard solution was not more than 1.2 (Table 9), and the USP plate count was more than 14200. It was found that the column temperature and pH of the mobile phase were crucial parameters and the flow rate of the mobile phase was less effective.

**Tab. 9:**
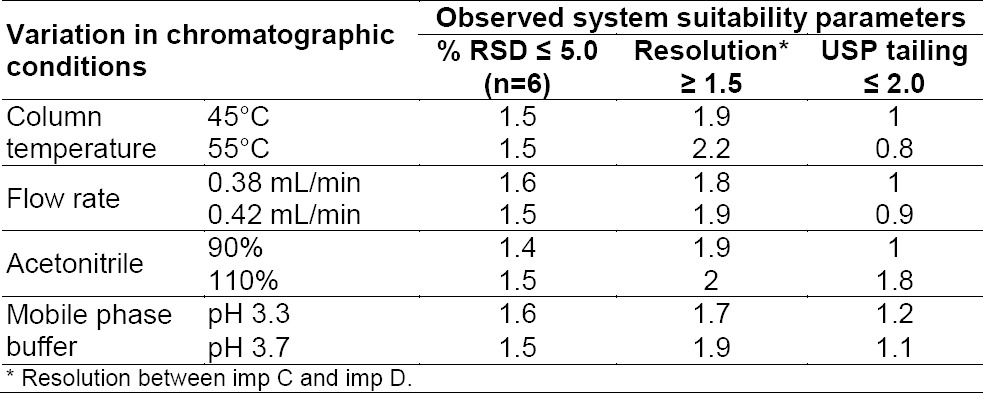
Robustness results of the UPLC methods

#### Stability of Solution and Mobile Phase

The variability in the estimation of all six impurities of tolterodine tartrate was within ±10% during the solution stability and mobile phase stability. The results from the solution stability and mobile phase stability experiments confirmed that the mobile phase was stable up to 48 hr and the sample solution and standard solutions were stable up to 48 hr.

## Conclusion

This research paper describes the identification and characterization of a degradant (des-*N*,*N*-diisopropyl tolterodine) in tolterodine tartrate pharmaceutical formulations. The impurity was isolated by semi-preparative liquid chromatography and characterized by using spectroscopic techniques. A simple and efficient RP-UPLC method development and validation were discussed. The method was found to be precise, accurate, linear, robust, and rugged during the validation. The method is stability-indicating and can be used for the routine analysis of production samples and to check the stability of the tolterodine tartrate drug product.
